# *Salvia verticillata* (L.)—Biological Activity, Chemical Profile, and Future Perspectives

**DOI:** 10.3390/ph17070859

**Published:** 2024-07-01

**Authors:** Stanislava Ivanova, Zoya Dzhakova, Radiana Staynova, Kalin Ivanov

**Affiliations:** 1 Department of Pharmacognosy and Pharmaceutical Chemistry, Faculty of Pharmacy, Medical University of Plovdiv, 4002 Plovdiv, Bulgaria; zoya.dzhakova@mu-plovdiv.bg (Z.D.); kalin.ivanov@mu-plovdiv.bg (K.I.); 2 Research Institute, Medical University of Plovdiv, 4002 Plovdiv, Bulgaria; 3 Department of Organisation and Economics of Pharmacy, Faculty of Pharmacy, Medical University of Plovdiv, 4002 Plovdiv, Bulgaria; radiana.staynova@mu-plovdiv.bg

**Keywords:** *Salvia verticillata*, *Salvia*, essential oils, folk medicine, phytopharmaceuticals, β-caryophyllene, salvianolic acid C, salvianolic acids

## Abstract

Species belonging to the genus *Salvia*, Lamiaceae, have been deeply involved in the folk medicine of different nations since ancient times. Lilac sage, or *Salvia verticillata* L. (*S. verticillata*) is a less studied species from the genus. However, it seems to have a prominent potential for the future drug discovery strategies of novel phytopharmaceuticals. This review aims to summarise the data on the biological activity and the phytochemical profile of extracts and essential oils derived from *S. verticillata*. This review is based on data from 57 in vitro and in vivo studies. The chemical profile of *S. verticillata* includes different synergic compounds like phenolic acids, flavonoids, terpenes, and salvianolic acids. Although some small amounts of salvianolic acid B were found in *S. verticillata* extracts, the major compound among the salvianolic acids is salvianolic acid C, a compound associated with the potential for improving liver fibrosis, cardio- and hepatoprotection, and the inhibition of SARS-CoV-2 infection. The cannabinoid type 2 receptor agonist β-caryophyllene is one of the major compounds in *S. verticillata* essential oils. It is a compound with a prominent potential in regenerative medicine, neurology, immunology, and other medical fields. The in vivo and the in vitro studies, regarding *S. verticillata* highlighted good antioxidant potential, anti-inflammatory, antibacterial, and antifungal activity. *S.verticillata* was also reported as a potential source of drug candidates for the treatment of neurodegenerative diseases such as Alzheimer’s disease, because of the inhibitory activity on the acetylcholinesterase. However, the number of studies in this direction is limited.

## 1. Introduction

The Lamiaceae family is one of the most widespread and diverse families in the plant world. It includes about 200 genera and over 3000 species of aromatic herbaceous plants, with a significant application in traditional medicine, cosmetics, the food industry, and the pharmaceutical industry as well.

The genus *Salvia* consists of over 900 species. The name of the genus comes from the Latin word “salvare”, meaning “to save” or “to heal” [1]. Species belonging to this genus have been used since ancient times as spices, repellents, diuretics, anti-inflammatory agents, and to improve fertility [2,3]. Nowadays, the *Salvia* species are commonly associated with anti-inflammatory, antibacterial, antifungal, antidiabetic, antioxidant, spasmolytic, and cytotoxic activity [4]. Several studies suggested that *Salvia officinalis* L. could affect Alzheimer’s disease. However, more detailed future studies are needed.

*Salvia verticillata* L. (*S. verticillata*), also known as lilac sage, is a worldwide perennial herbaceous plant that is native to western Asia and eastern and central Europe. *S. verticillata* grows in a continental climate characterised by a moderate amount of rainfall [5]. It is a drought-tolerant plant that grows in warmer temperatures and its development is favourable under sufficient conditions of heat and moisture. Higher temperatures contribute to an increase in essential oil yield, while moisture decreases it [6]. *S. verticillata* is a semi-shrub, reaching a height of up to 50 cm. The stems are annual, ascending, branched, mossy, and about 1.5 cm thick. The leaves can be heart-shaped or ovate-triangular in shape, simple, pointed, and also mossy. The inflorescences are branched and reach a height of about 25 cm [5]. The flowers of *S. verticillata* are large and violet, bisexual, and bilobed. They are arranged in axillary or terminal inflorescences [7]. The seeds are ovoid, slightly elongated, smooth, and light brown. The cultivation of *Salvia* requires an altitude of up to 1000 m and protection from cold and moisture. The plants are cross-pollinating and flower in the second year in the summer [6]. The flowering period could continue from May to September [7].

*S. verticillata* is a plant with promising potential for the drug-discovery strategies of new therapeutic agents. Despite the wide distribution and use of *Salvia* species in traditional medicine, more in vitro and in vivo studies are needed to analyse the biological activity of *S. verticillata* and to establish the therapeutic potential of the herb. Currently, there is a lack of randomised clinical trials.

Although the synthesis of novel molecules is deeply involved in the modern pharmaceutical industry, plants might play a crucial role in future drug discovery strategies [8,9,10,11,12,13,14,15,16,17,18].

The purpose of this research is to review the chemical profile of *S. verticillata*, to sum up the data of the in vivo and in vitro studies, and to summarise *S. verticillata’s* biological activity studies. This study highlights the relationship between the chemical profile of *S. verticillata*, the biological activity, and future perspectives.

## 2. Results and Discussion

### 2.1. Chemical Composition of Salvia Extracts and Essential Oils

The chemical composition of plant extracts and essential oils (EOs) isolated from the same species could deeply vary depending on the plant origin, the plant organ, the development stage, and the ecological and climatic factors. The methods of extraction and the drying processes also influence the chemical profile [19]. The literature data on *S. verticillata* reveal considerable variation related to the EO constituents, and chemovariability is observed at the species and subspecies levels. The composition of *S. verticillata* oil and extracts deeply varies in the different geographical regions. The main groups of secondary metabolites of *S. verticillata* are phenolic compounds, such as phenolic acids and flavonoids, and terpenes—monoterpenes and diterpenes [1].

Extracts isolated from the aerial parts of *S. verticillata* species contain polyphenols such as caffeic acid, rosmarinic acid, and the major metabolites salvianolic acids, as well as the flavonoid apigenin and its 7-*O*-derivatives [1,20,21,22,23,24] (Table 1).

Rosmarinic acid is one of the major compounds found in most of the *S. verticillata* extracts. In general, the levels of rosmarinic acid are highly variable and normally are between 0.15 and 41.53 mg/g [23]. The compound is associated with diverse biological activity like antibacterial, antifungal, antiviral, antioxidant, anticancer, anti-inflammatory [25], hepatoprotective, cardioprotective, nephroprotective, antidiabetic, antiallergic, anti-ageing, and antidepressant activity [26].

Salvianolic acids, which were identified in extracts from Serbia, Iran, and Turkey, are regarded as compounds with significant antioxidant and neuroprotective activity. Recently, it has been discussed the potential benefits of these compounds in the management of neurodegenerative diseases such as Parkinson’s disease [27]. In vitro, hepatoprotective activity was also established [28].

The flavone apigenin, which is also regarded as one of the main compounds of *Salvia* extracts, is associated with antidiabetic activity, anticancer activity [29], anti-inflammatory activity, neuroprotective activity, and antioxidant effects. The compound is studied as a potential agent to slow the progression of Alzheimer’s disease [29].

The majority of the examined EOs of *S. verticillata* from Iran are characterised by the presence of β-caryophyllene, muurolene, α- and β-pinene, germacrene D, β-phellandrene, α-humulene, 1,8-cineole, and spathulenol. EOs of *S. verticillata* from Turkey contain mainly spathulenol, limonene, α- and β-pinene, germacrene D, and 1,8-cineole. The main volatile compounds identified in the Greek *S. verticillata* EOs are α- and β-pinene. Two different chemical profiles of Serbian *S. verticillata* EOs have been observed, one of which contains high levels of β-phellandrene and myrcene [30], while the other is characterised by the presence of high levels of germacrene D and E-caryophyllene [31] (Table 2).

For targeting the possible therapeutic strategies for the implementation of *S. verticillata* EOs in the contemporary pharmaceutical industry and performing more profound studies on the biological activity, it is essential to consider the full chemical profile of the EOs rather than focusing solely on the individual compounds. The possible interactions between the different compounds in the compositions of EOs are also important. The phytochemical profile of *Salvia* EOs suggested a great potential for future application of EOs in neurology and in endocrinology as a hypoglycaemic agent in the management of diabetes.

For example, the natural bicyclic sesquiterpene β-caryophyllene, which was found in the samples from Italy, Iran, and Romania (in concentrations between 11.9% and 31.5%), has a significant potential to be used in the prevention and treatment of different neuroinflammatory and neurodegenerative pathologies [48]. In general, the compound is safe. According to the Organization for Economic Cooperation and Development guidelines, β-caryophyllene is regarded as toxic at doses greater than 2000 mg/kg [48,49]. Studies on acute toxicity reported that oral administration of 2000 mg/kg of β-caryophyllene in female mice induced no toxic effects [48]. In general, *S. verticillata* EOs seem to be one of the best sources of β-caryophyllene. Although the compound could be isolated from the EOs of a wide variety of plants, its concentrations in *S. verticillata* EOs are one of the highest. Other plants which are considered rich sources of the compound are *Cinnamomum tamala* (concentration of about 25.3% in the EO) and *Cannabis sativa* (concentration in the EOs between 3.8 and 37.5%) [13].

The compound is regarded as a dietary cannabinoid. More precisely, it is a cannabinoid type 2 receptor agonist [11].

**Figure 1:**
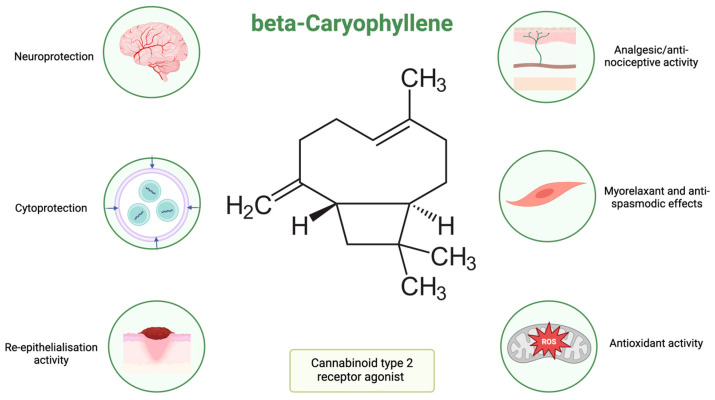
Β-caryophyllene—structure and biological activity (created with BioRender.com, assessed on 27 May 2024).

Many in vivo studies reported that β-caryophyllene (Figure 1) reduced/inhibited the activity of nitric oxide synthase and improved the activity of the antioxidant enzymes, affecting lipid peroxidation, as well as glutathione levels [50,51].

β-Caryophyllene is also associated with inhibition of the expression of IL-6 and IL-1β, which are also related to the inflammatory process, by stimulating cytokine production [13]. At concentrations of 10, 25, and 50 μM, β-caryophyllene was reported to reduce the levels of nitric oxide and prostaglandin E2, as well as suppress the nuclear factor kappa B (NF-κB) activation on BV2 mouse cell lines, reducing neuroinflammation [48]. Neuroprotection seems to be one of the target directions for this molecule. It was reported that at doses of 5 mg/kg, β-caryophyllene exerts nonpsychoactive anti-inflammatory effects [13]. It was established that emulgel formulation containing 1% β-caryophyllene promotes the wound healing processes in animals [52]. The authors reported that the treatment with β-caryophyllene enhanced the re-epithelialisation and increased laminin-γ2 and desmoglein-3 immunolabeling [52].

A recent study reported that supplementation with β-caryophyllene may have beneficial effects on obese individuals [11]. Animal studies reported that the intake of the molecule had significant effects on weight reduction (including a significant reduction in body fat %), improvement of dyslipidaemia, improved steatosis and ballooning of liver cells, and reduced adipogenesis [53,54].

Although *S. verticillata* EOs were not yet tested for wound healing activity, it seems that *S. verticillata* EOs could provide many benefits in skin recovery. Both β-caryophyllene and its isomer α-humulene are widely distributed in the composition of EOs of various plants including *S. verticillata.* α-Humulene, also known as α-caryophyllene, is a monocyclic sesquiterpene primarily isolated from the essential oil of *Humulus lupulus*. Experimental studies on α-humulene and its isomers highlighted significant anti-tumour potential and cytotoxic activity against cancer cells, effectiveness against a wide range of microorganisms, and anti-inflammatory and analgesic activity. These compounds were reported to provide good gastroprotective and antioxidant activity as well [55]. The sesquiterpenes α-humulene and β-caryophyllene were found to be synergistic with doxorubicin and at the same time do not have cytotoxic effects on normal cells [55]. One of the mechanisms is related to the elevation of the level of reactive oxygen species (ROS) in the mitochondria, which leads to a decreased membrane potential. Calcium is released into the cytosol, and this can lead to cellular imbalance and cell death [56,57,58]. On the other hand, cell apoptosis can be induced by increasing the activity of enzymes caspase 3 and caspase 8 (which can be triggered by TNF), as well as by the elevation of ROS levels, which stimulate receptors on the surface of cells (DR4/DR5) [59,60,61,62]. It also has anti-inflammatory activity due to influencing factors of the inflammation process, such as NF-κB, which controls cytokine production. A study demonstrated the antibacterial activity of α-humulene at a dose of 2 μg/mL on strains WT-ETBF, rETBF, and WT-NTBF of *Bacteroides fragilis*, as well as inhibiting biofilm formation [63]. The antibacterial activity of α-humulene and germacrene D is due to an effect to a lesser extent on Gram-positive and to a greater extent on Gram-negative microorganisms. α-Humulene has potential analgesic, antioxidant, and gastroprotective effects [55,64].

The monoterpenes α- and β-pinene are other major constituents of *S. verticillata* EOs. These bicyclic structural isomers, widely present in the composition of many other plants, are associated with diverse biological effects, such as antimicrobial, antiviral, analgesic, anti-inflammatory, antioxidant, and fungicidal activity [65]. It was established in vitro that α-pinene reduces the synthesis of thromboxane A2 and suppresses the platelet aggregation [66]. α-Pinene was also reported to provide good anticancer activity on human cell lines from liver cancer, ovarian cancer, and neuroblastoma. A significant synergism was observed between α- and β-pinene and Paclitaxel in non-small-cell lung cancer cells [65,67]. Neuroprotection, suppression of the formation of ROS, and enhancement of the expression of enzymatic antioxidants were also reported [65,68]. α- and β-pinene were also found to exhibit anti-epileptic activity. In a study with mice, α-pinene, β-pinene, and a mixture containing both of the compounds were administered in doses of 100–400 mg/kg, as well as diazepam in a dose of 2 mg/kg. After 1 h, the mice were injected with pentylenetetrazole to induce convulsions, and a significant reduction in seizure intensity was observed [65,69].

Other major compounds found in *S. verticillata* EOs are the germacrenes and sesquiterpenes, which are involved in the synthesis of other sesquiterpenes. It is considered that the name “germacrene” origins from “germacrone”, a compound isolated for the first time from *Geranium macrorrhizum* oil [70]. The germacrenes are associated with antioxidant, anti-inflammatory, antifungal, anticancer, and insecticidal activity [70,71].

β-Phellandrene, a monocyclic monoterpene, is present in *S. verticillata* EOs from Iran, Serbia, Turkey, and Italy. It is known as an insecticide of natural origin, with such activity proven by some studies. One of the in vivo studies performed with β-phellandrene demonstrated significant genotoxicity at a dose of 1425–2850 mg/kg. Other studies do not prove such but still consider possible damage to the DNA strands [72].

The monoterpene 1,8-cineole, or eucalyptol, which can be isolated from the EOs of *Eucalyptus*, as well as from *Salvia* and *Melaleuca quinquenervia*, is widely used in the cosmetic and perfume industry. The compound is included in the composition of repellents. Eucalyptus has demonstrated significant antioxidant and anti-inflammatory activity, inhibiting the synthesis of ROS and inflammatory cytokines [73,74]. The compound has a therapeutic potential for the management of some cardiovascular, respiratory, and digestive diseases. It was studied in the treatment of bronchitis, asthma, rhino sinusitis, pneumonia, flu, and other conditions. The intake of the compound is associated with anti-inflammatory effects and muscle relaxation and affects mucus hypersecretion by affecting interleukin-1b and tumour necrosis factor-a (TNF-a). It is considered that the anti-inflammatory effect might be a result of binding to the NF-jB [73]. In vitro and in vivo anticancer activity studies demonstrate the potential for the use of 1,8-cineole in the treatment of breast, ovarian, liver, skin, and colon cancer. It was established that the compound induces tumour cell apoptosis through tumour suppressor protein p53 [75]. COX-2-induced expression of the aryl hydrocarbon receptor (AhR) was also reported to be suppressed [76]. Moreover, 1,8-cineole exhibits analgesic, anaesthetic, sedative, antifungal, and antimicrobial effects, affecting *Escherichia coli*, *Staphylococcus aureus*, *Pseudomonas aeruginosa*, and *Bacillus subtilis*. The target of 1,8-cineole is L-asparaginase, yet its antimicrobial activity alone is relatively low. This activity is enhanced when applying oil, due to the synergism of the compounds in the composition. Synergism has also been observed between chemical agents with antibacterial activity, such as mupirocin and 1,8-cineole, but the mechanisms of action are not fully established [73].

The levels of spathulenol, in *S. verticillata* EOs could reach about 17%. In general, the concentrations of this tricyclic sesquiterpenoid are between 5 and 9%. Currently, spathulenol is regarded as a compound with significant antioxidant potential and antibacterial, antifungal, antiseptic, anti-nociceptive, and antitumour activities. It is used in the management of diabetes, rheumatoid arthritis, and wound healing. An in vitro study demonstrated the protective activity of spathulenol on 6-hydroxydopamine-treated neuroblastoma cells. 6-OHDA (6-hydroxydopamine) stimulates the formation of catecholamine quinones and ROS, which induce oxidative stress and cell death [77]. The combination of 6-OHDA and spathulenol resulted in a dose-dependent recovery of damaged cells. Therefore, it can be used in the treatment of neurodegenerative diseases [77]. In an in vitro study, spathulenol demonstrated activity on mice lymphoma cell lines. It has been proven that spathulenol can be used as an adjuvant in anticancer therapy, but in vivo studies are also needed [78].

The composition of extracts differs not only by region and country but also by the solvents used in the process. Sometimes variations in the concentration of a substance in the composition are obtained precisely because of this. Other factors are the time of picking, cultivation, and conditions for nourishing the plant.

The metabolites, from both extracts and EOs, are responsible for antioxidant, anti-inflammatory, antibacterial, cytotoxic activity, etc. [21].

A significant number of in vitro and less in vivo studies on *S. verticillata* reported activities and highlighted the therapeutic potential of the plant. However, more in vivo studies are needed.

### 2.2. Biological Activity

**Figure 2:**
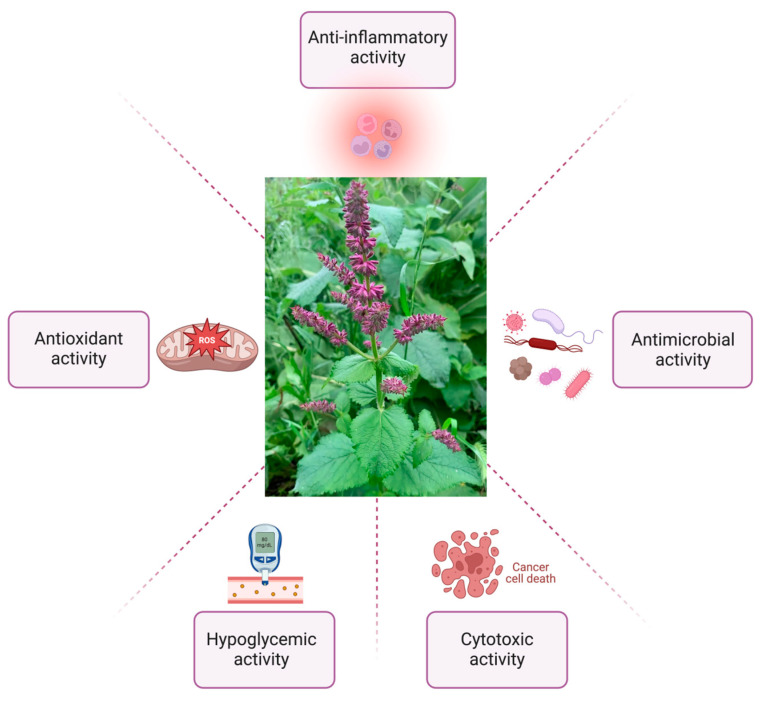
Biological activity of *Salvia verticillata* (created with BioRender.com, assessed on 26 May 2024).

The biological activity of *S. verticillata* is mainly due to two main groups of secondary metabolites from the chemical composition of the plant: phenolic compounds (phenolic acids, flavonoids) and terpenes (monoterpenes and diterpenes). A high percentage of the phytochemical composition is represented by terpenoids such as caryophyllene, camphor, and α-thujone; and polyphenols such as caffeic acid, rosmarinic acid, and their metabolites, yunnaneic and salvianolic acids. Also, flavonoids like luteolin, apigenin and its 7-*O*-derivatives, cirismaritin, and quercetin were reported [1]. *Salvia* species roots mainly contain diterpenes, while the aerial parts—flowers and leaves—contain monoterpenes, triterpenoids, and flavonoids. These metabolites are responsible for antioxidant, anti-inflammatory, antibacterial, cytotoxic activity, etc. (Figure 2) [21].

The high levels of rosmarinic acid in the composition of *S. verticillata* extracts are responsible for the significant antibacterial activity and milder antifungal activity. Other compounds, such as luteolin, apigenin, quercetin, and diterpenoids such as carnosic acid and carnosol, contain in their molecules several hydroxyl (OH) groups, which bind to the enzymatic active centre of microorganisms and suppress their growth [1]. EOs from *S. verticillata* have higher antibacterial activity as they contain substances such as limonene, α-pinene, β-pinene, α-thujene, and myrcene [1].

The antibacterial activity of *S. verticillata* has been demonstrated by several in vitro studies on both extracts and EOs.

It was reported that *S. verticillata* extracts provide antibacterial activity against *Escherichia coli*, *Pseudomonas aeruginosa*, *Salmonella enteritidis*, etc. [39,79,80] (Table 3).

The antioxidant activity of *S. verticillata* extracts is comparable to that of *Salvia officinalis* (Table 3) [22,87,91,93]. These data point out the use of *S. verticillata* as a natural antioxidant in the food industry. The antioxidant capacity of *Salvia* extracts is mainly due to phenolic compounds, and especially rosmarinic acid. These compounds act as donors of hydrogen atoms and affect free radicals in different phases, for example, the distribution, initiation, and activation of enzymes with antioxidant activity [1]. The antioxidant capacity of *S. verticillata* extracts is comparable to that of the essential oil.

*S. verticillata* extracts are also sources of salvianolic acid C, which reduces the free radical levels. Currently more than 10 different salvianolic acids were identified: salvianolic acid A, B, C, D, E, F, G, etc. These water-soluble compounds are specific for the *Salvia* species [101]. Salvianolic acids are associated with different biological activities, including multiple mechanisms for cardiovascular protection [101], anticancer activity [102], etc.

**Figure 3:**
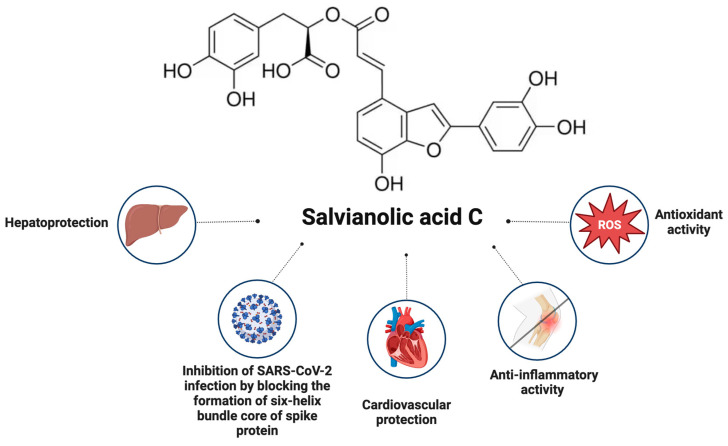
Biological activity of salvianolic acid C (created with BioRender.com, assessed on 27 May 2024).

In 2020, it was reported that salvianolic acid C could inhibit SARS-CoV-2 infection by blocking the formation of the six-helix bundle of the core of the spike protein [103]. The compound is also associated with being a cardioprotector [101] and hepatoprotector [104] and as a compound with a potential for improving liver fibrosis [102] (Figure 3).

The hepatoprotective activity of salvianolic acid C was tested in acetaminophen overdose in mice [104]. It was reported that salvianolic acid C can prevent the elevation of the serum biochemical parameters and the lipid profile including aspartate aminotransferase, alanine aminotransferase, and total bilirubin. The study provided significant evidence that salvianolic acid C can protect the hepatocytes from acetaminophen-induced damage by mitigating mitochondrial oxidative stress and the inflammatory response and can be mediated by the caspase anti-apoptotic effect through the inhibition of the Kelch-like ECH-associated protein 1/erythroid 2-related factor 2/heme oxygenase-1 signalling axis [104].

Recently, the compound gained attention as a potential molecule that could be involved in the therapy of the early phase of ischemic stroke [105]. Wenbo Guo and colleagues reported that treatment with salvianolic acid C can significantly reduce the infarct volume, improve the neurological deficits, and reverse the pathological changes in the transient middle cerebral artery occlusion in mouse models [105]. Salvianolic acid C was isolated also from other *Salvia* species, such as *Salvia miltiorrhiza*.

The therapeutic potential of salvianolic acid C could be quite diverse. Moreover, a synergism exists between the beneficial effects of salvianolic acid C and the other compounds found in the composition of *S. verticillata* extracts. In the next decades, it is highly likely for this compound to be included in the composition of novel drug candidates with target oncology, neurology, cardiology, and gastroenterology.

In vitro studies reported that the EO affects both Gram-positive and Gram-negative microorganisms [106,107], affecting mainly *Escherichia coli*, with almost no activity on *Pseudomonas aeruginosa* [2,46] (Table 4).

*S. verticillata* EOs and its extracts are associated with significant antioxidant activity comparable to that of *Salvia officinalis* [81,91]. The antioxidant activity varies according to the solvents used in the extraction process. According to Katanić Stanković, methanolic extracts of *S. verticillata* showed mild antimicrobial properties [1]. It was reported that *S. verticillata* has significant antibacterial activity, especially against *Escherichia coli* and *Staphylococcus aureus* [39,41], and has no effect on *Pseudomonas aeruginosa* [2,46]. α-Pinene found in the EO of *S. verticillata* demonstrates higher activity against strains of *Escherichia coli* compared to that of β-pinene and 1,8-cineole. These compounds do not show activity against the bacterial strains of *Pseudomonas aeruginosa* [2]. *Pseudomonas aeruginosa* also shows resistance to *S. verticillata* EO from Iran, the main constituents of which are β-caryophyllene and germacrene D [46]. Antifungal activity was also established against *C. albicans* [2,106], *C. glabrata*, and *Saccharomyces cerevisiae* [106]. According to Kunduhoğlu, the EO has inhibitory activity against butylcholinesterase and acetylcholinesterase [106]. Although the research is scarce, *S. verticillata* may be used as a neuroprotectant. One of the in vitro studies tested *S. verticillata* essential oil from Georgia for anti-inflammatory activity, demonstrating such activity [108].

Several in vivo studies investigated the biological activity of *S. verticillata* (Table 5). The main focus of these studies was the evaluation of the antioxidant potential, evaluation of the hepatoprotection, and evaluation of the anti-inflammatory activity [4,109,110,111,112].

In vivo studies on *S. verticillata* are quite limited. The main focus of these studies is the antioxidant [109,111] and the anti-inflammatory activities [4,112]. A study on the hypoglycaemic activity of *Salvia* showed that the extract significantly affected diabetic mice and had hepatoprotective activity [110].

### 2.3. Future Potential for Use of Salvia verticillata

There are few in vitro and in vivo studies, proving the neuroprotective effect of *Salvia*, specifically *S. verticillata* [94,98,100]. The intake of *S. verticillata* extracts was associated with beneficial effects on the learning process, memory, and attention. This suggests that *S. verticillata* could be used as an adjunctive therapy in neurodegenerative diseases, including Alzheimer’s disease [113]. Future research may focus specifically on the chemical composition of the plant and more thoroughly explore its potential for slowing the progression of these diseases. Moreover, the EOs isolated from *S. verticillata* contain the cannabinoid type 2 receptor agonist β-caryophyllene, a compound with a prominent potential in the management of Alzheimer’s disease [13].

Alzheimer’s disease, which is an important challenge for contemporary medicine and science, is characterised by increased activity of the enzyme acetylcholinesterase, resulting in low synaptic cleft levels of acetylcholine. The disease has a negative effect on cognitive functions, with low levels of acetylcholine leading to impaired memory, concentration, motivation, and the ability to learn [100]. *Salvia* constituents such as monoterpenes, phenolic diterpenes, quercetin, and rosmarinic acid show inhibitory activity against acetylcholinesterase [113]. One of the in vitro and ex vivo studies used isolated guinea pig ileum, knowing that acetylcholine causes a concentration-dependent contraction. *Salvia* extracts demonstrated a slight dose-dependent effect on acetylcholinesterase inhibitory activity [100].

Another in vitro study compared the acetylcholinesterase and butylcholinesterase inhibitory activity of a petroleum ether extract of *S. verticillata* and Galantamine. The results showed relatively good concentration-dependent inhibitory activity [89].

The ability of *S. verticillata* to slow down the progression of the disease should be studied more profoundly. Currently, Alzheimer’s disease remains an unsolved challenge, and the treatments focus mainly on the management of the symptoms and improving the quality of life.

Several studies investigated the hypoglycaemic activity of *S. officinalis*. One in vivo study investigated the effects of the methanolic extract and essential oil of *S. officinalis* leaves on rats with diabetes induced by streptozotocin injection. A significant increase in glucose levels was observed only after five days. The results showed that *Salvia* methanolic extract, unlike the essential oil, provided hypoglycaemic activity [114]. This and many other studies could be a good basis for future research on the hypoglycaemic activity not only in *S. officinalis* but also in other species like *S. verticillata*.

Another important in vivo study demonstrated that the intake of ethanolic extract of *S. verticillata* for 14 days resulted in a decrease in the glucose levels, and a positive influence and protective effects on the liver and kidneys [110].

Some in vitro clinical studies have focused on the cytotoxic activity of *S. verticillata*, but their number is limited. One of them used essential oil from the leaves and flowers of *S. verticillata* and colorectal carcinoma and breast ductal carcinoma cell lines and embryo fibroblast from rats. The cytotoxic activity of the essential oil has been proven, with the greatest effect on colorectal adenocarcinoma [37].

The chemical profile of *S. verticillata* EOs highlights a potential for implementation in regenerative medicine. The main compounds isolated from the EOs were already reported to promote the wound healing processes. These effects in addition to the mild antibacterial and antifungal activity make the EO worth studying as a skin recovery therapy after surgical procedures, dermatological conditions, or other interventions [115,116,117,118]. Currently, there are no human or animal studies investigating the skin recovery effects of EOs isolated from *S. verticillata*.

## 3. Materials and Methods

**Figure 4:**
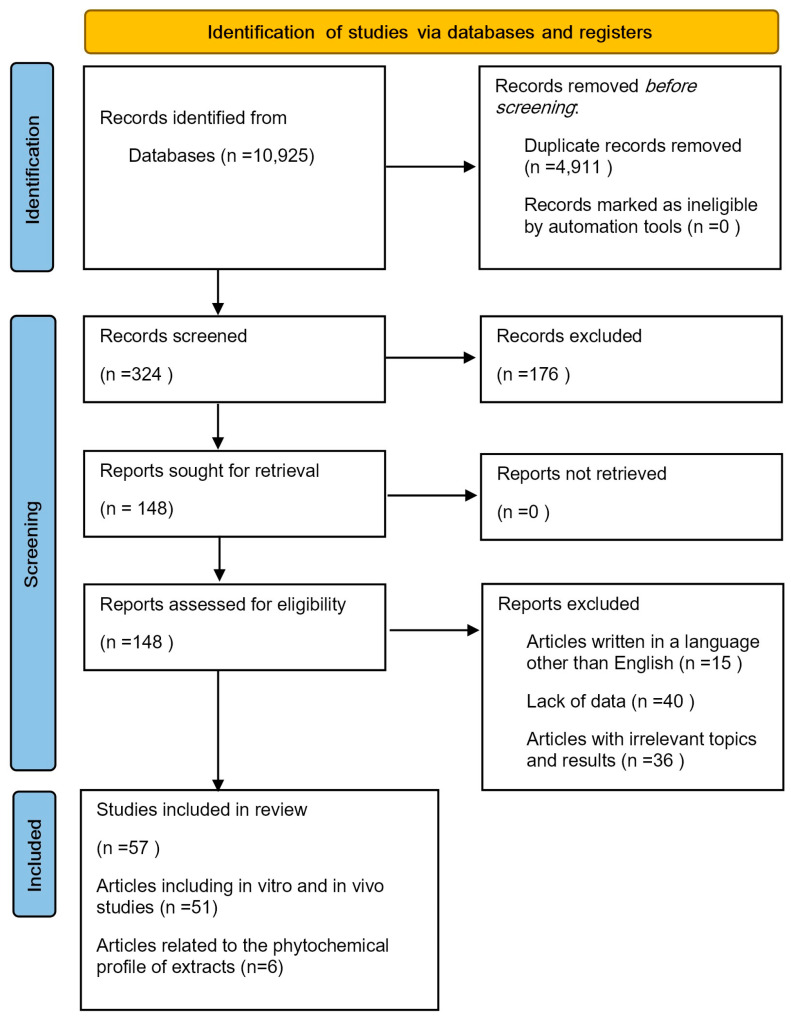
PRISMA 2020 flow diagram [119].

The first step of the screening process involved identifying eligible studies as well as following the Preferred Reporting Items for Systematic Reviews and Meta-Analyses (PRISMA) guidelines (Figure 4) [119]. An extensive search of the PubMed, Scopus, Web of Science, and Google Scholar databases was performed.

The following keywords were used in the search process: “*Salvia verticillata*”, “*Salvia verticillata* essential oil study”, “*Salvia verticillata* antioxidant activity”, “anticancer properties of *Salvia verticillata*”, “*Salvia verticillata* composition”, “Chemical composition of *Salvia verticillata”*, “*Salvia verticillata* and Alzheimer’s disease”, and “*Salvia verticillata* antibacterial activity”. In the final stage, relevant studies were selected based on the exclusion and inclusion criteria. The exclusion criteria were webinars or blogs, and articles containing a lack of data. The inclusion criteria were in vitro studies, animal studies, and studies investigating the impact of the chemical composition. A total of 57 studies on the biological activity of *S.verticillata* extracts and EOs were selected and included in the present review.

## 4. Conclusions

*Salvia verticillata* is a plant species which could play a crucial role in the future drug discovery strategies. Although the plant is associated with deep traditions in the folk medicine of different nations, studies that investigated its therapeutic potential are limited. The chemical profile of *S. verticillata* includes different compounds like phenolic acids, flavonoids, terpenes, and salvianolic acids. In the last decade, salvianolic acids were reported to provide different biological activities, including multiple mechanisms for cardiovascular protection, anticancer activity, etc. Although some small amounts of salvianolic acid B were found in *S. verticillata* extracts, the major compound among the salvianolic acids was salvianolic acid C, a compound associated with the potential for improving liver fibrosis, cardio- and hepatoprotection, the inhibition of SARS-CoV-2 infection by blocking the formation of the six-helix bundle of the core of the spike protein, and anti-inflammatory activity.

*S. verticillata* EOs seem to be rich sources of the cannabinoid type 2 receptor agonist β-caryophyllene, a compound with a prominent potential in a wide variety of medical fields. The in vivo and the in vitro studies regarding *S. verticillata* extracts and EOs highlighted significant antioxidant, anti-inflammatory, antibacterial, and antifungal activity. Recently, *S. verticillata* has been reported as a potential agent for the treatment of neurodegenerative diseases such as Alzheimer’s disease because of its inhibitory activity on acetylcholinesterase. However, the number of studies in this direction is limited. *S. verticillata* extracts could be also used in the food industry as novel food additives to slow the oxidation processes in meat or other food products.

## Figures and Tables

**Table 1:** Phytochemical profile of *S. verticillata* extracts.

Plant Collecting Region	Plant Materials	Collecting Period	Compounds	References
Serbia	Aerial parts (methanol extract)	July	Rosmarinic acid, Salvianolic acid C, Rosmarinic acid hexoside, Methylrosmarinate, Salvianolic acid B, Dicaffeoylquinic acid, 5-*O*-Caffeoylquinic acid, Caffeic acids, Apigenin-7-*O*-glucosides, Apigenin, Quercetin 3-*O*-rutinoside, Carnosol, Carnosic acid, Quercetin 3-*O*-rhamnoside	[1]
Turkey	Aerial parts (methanol extract)	August	Rosmarinic acid	[20]
Turkey	Aerial parts (dichloromethane, methanol, aqueous extracts)	No data	Rosmarinic acid, Salvianolic acid C derivative, Apigenin-*O*-glucuronide, Luteolin-*O*-glucuronide, Salvianolic acid K, Methyl rosmarinate, Caffeoyl hexoside, Danshensu	[21]
Romania	Aerial parts (methanol extracts)	July	Rosmarinic acid, Apigenol, Caffeic acid, Chlorogenic acid, Luteolin, *p*-Coumaric acid	[22]
Iran	Leaves, roots (methanol extracts)	June–July	Rosmarinic acid, Salvianolic acid B, Salvianolic acid A, Carnosic acid, Caffeic acid	[23]
Turkey	Root, leaf, stalk, flower (methanolic extracts)	June	Kaempferol, Catechin, Quercetin, Myricetin, Naringenin, Resveratrol, Naringin, Rutin	[24]

**Table 2:** Composition of main volatile compounds in *Salvia verticillata* EOs.

Plant Collecting Region	Plant Materials	Collecting Period	Main Volatile Compounds	Other Volatile Compounds	References
Iran	Aerial parts	May–June	β-Caryophyllene (24.7%), γ-Muurolene (22.8%), Limonene (8.9%), α-Humulene (7.8%), Germacrene B (6.6%), β-Pinene (5.1%).	α-Gurjunene (4.9%), Sabinene (2.7%), α-Pinene (2.6%), Myrcene (1.9%), (*Z*)-β-Ocimene (1.4%), (*E*)-β-Ocimene (1.4%), β-Borbonene (0.9%), α-Phellandrene (0.8%), Alloaromadendrene (0.7%), Caryophyllene oxide (0.6%), α-Copaene (0.5%), α-Thujene (0.3%), δ-3-Carene (0.3%), α-Cubebene (0.3%), β-Cubebene (0.3%), β-Cadinene (0.3%), δ-Cadinene (0.3%), Camphene (0.2%), γ-Terpinene (0.2%).	[32]
Turkey	Aerial parts (flowers, leaves, stems)	July	Spathulenol (31.0%), α-Pinene (8.2%).	Limonene (4.1%), Hexahydrofarnesyl acetone (3.8%), Caryophylla-2(12), 6-dien-5β-ol (=Caryophyllenol I) (2.8%), 1,8-Cineole (2.5%), Hexadecanoic acid (2.3%), Linalool (2.2%), Carvacrol (2.1%), β-Pinene (2.0%), Caryophylla-2(12),6-dien-5β-ol (=Caryophyllenol II) (2.0%), Caryophyllene oxide (1.9%), Clovenol (1.8%), Caryophylla-2(12),6(13)-dien-5β-ol (=Caryophylladienol II) (1.8%), Myrcene (1.4%), γ-Terpinene (1.1%), *p*-Cymene (1.0%), α-Terpineol (1.0%), Humulene epoxide-II (0.9%), Verbenone (0.8%), β-Caryophyllene (0.7%), T-Cadinol (0.7%), α-Phellandrene (0.6%), Camphor (0.6%), Terpinen-4-ol (0.6%), Citronellol (0.6%), Globulol (0.6%), Viridiflorol (0.6%), α-Cadinol (0.6%), Hexanal (0.5%), Torilenol (0.5%), Eudesma-4(15),7-dien-4β-ol (0.5%), Borneol (0.4%), Salvial-4(14)-en-1-one (0.4%), Thymol (0.4%), Bornyl acetate (0.3%), *trans*-Pinocarveol (0.2%), *cis*-Piperitol (0.2%), Carvone (0.1%).	[33]
Greece	Aerial parts (leaves, inflorescences)	August	β-Pinene (30.7%), *p*-Cymene (23.0%), Isopropyl ester of lauric acid (16.8%), α-Pinene (7.6%), (*E*)-Nerolidol (5.2%).	1,8-Cineole (3.9%), γ-Terpinene (1.9%), α-Copaene (1.9%), β-Bourbonene (1.7%), α-Thujene (1.3%), *trans*-Calamenene (1.2%), γ-Muurolene (1.1%), *cis*-Muurola-3,5-diene (1.0%), *trans*-Pinocarveol (0.8%), β-Gurjunene (0.7%), Heptadecane (0.7%).	[34]
Serbia	Aerial parts (flowers, leaves) Comparison between 3 populations of EOs.	No data	Pop. 1: Germacrene D (48.0%), (*E*)-Caryophyllene (13.4%), α-Cadinol (10.4%), α-Humulene (7.2%), δ-Cadinene (6.0%), Eudesma-4(15), 7-dien-1-beta-ol (6.0%), Bicyclogermacrene (5.3%). Pop. 2: Germacrene D (24.6%), (*E*)-Caryophyllene (19.0%), Bicyclogermacrene (16.7%), α-Humulene (10.2%), β-Phellandrene (8.6%), (*E*)-β-Ocimene (7.5%), Spathulenol (7.2%), (*Z*)-β-Ocimene (6.0%). Pop. 3: (*E*)-Caryophyllene (10.2%), β-Cubebene (8.6%), Eicosane (8.5%), Spathulenol (6.5%).	Pop. 1: Spathulenol (3.5%). Pop. 3: α-Humulene (4.8%), Cyclopentane (4.5%), Naphthalene, 1, 2, 3, 4, 4a, 5, 6, 8a-octahydroxyl (4.0%), δ-Cadinene (3.7%), α-Cadinol (3.7%), Caryophyllene oxide (2.9%), 2-Pentadecanone, 6, 10, 14-trimethyl (2.5%), 1,2-benzenedicarboxylic acid (2.5%), Aromadendren epoxide (2.1%), β-Phellandrene (1.7%), γ-Cadinene (1.3%), Naphthalene, 1, 2, 3, 4, 4a, 7-hexahydroxyl (1.3%), β-Elemene (1.1%), α-Elemene (1.1%), α-Muurolene (1.0%), γ-Gurjunene (0.9%), Camphene (0.6%), Caryophyllenol-11 (0.6%), α-Pinene (0.5%), α-Cubebene (0.5%), Octacosane (0.5%), Nonahexacontanoic acid (0.5%), β-Bourbonene (0.4%).	[31]
Turkey	Aerial parts	June	Germacrene D (13.8%), Spathulenol (10.0%).	Limonene (4.5%), 1,8-Cineole (4.0%), β-Copaene (3.8%), Bicyclogermacrene (3.3%), Naphthalane (3.1%), δ-Cadinene (2.9%), β-Pinene (2.8%), α-Pinene (2.7%), Valeranone (2.5%), Sabinen (2.1%), β-Bourbonene (2.0%), α-Cubebene (1.9%), β-Caryophyllene (1.8%), α-Copaene (1.7%), β-Cubebene (1.7%), Caryophyllene oxide (1.7%), α-Eudesmol (1.7%), Cyercene (1.7%), Salvial-4 (14)-en-1-one (1.5%), 2-Pentadecanone (1.5%), δ-3-Carene (1.3%), Methyl ısoeugenol (1.3%), γ-Cadinene (1.3%), *cis*-Calamenene (1.3%), (+)-Epi-bicyclosesquiphallendrene (1.2%), Eudesma-4 [15], 7-dien-1-beta-ol (1.2%), *p*-Cymene (1.1%), β-Myrcene (0.9%), Sabinene (0.9%), δ-Selinene (0.9%), *trans*-β-Farnesene (0.8%), Jasmone (0.8%), Isolongifolene (0.8%), Epi-α-Cadinol (0.7%), Cadalene (0.7%), Ethanone (0.7%), Borneol-L (0.6%), Aromadendrene (0.6%), 5,9-Undecadien (0.6%), Nerolidol (0.6%), γ-Gurjunene (0.6%), Muurola-3,5-diene (0.5%), α-Calacorene (0.5%), *trans*-Verbenol (0.4%), α-Terpineol (0.4%), α-Ylangene (0.4%), α-Amorphene (0.4%), β-Selinene (0.4%), α-Thujone (0.3%), Benzeneacetaldehyde (0.3%), α-Cadinene (0.3%), Vulgarol-B (0.3%), Camphene (0.2%), Mentha-1 (7), 8-diene (0.2%), Benzene, 1-methyl-2 (0.2%), γ-Terpinene (0.2%), *cis*-Sabinenehydrate (0.2%), Pinocarvone (0.2%), 3-Cyclohexen-1-ol (0.2%), *trans*-Carveol (0.2%), Propanol, 2-methyl-3-phenyl (0.2%), Bornyl acetate (0.2%), Humulene epoxide II (0.2%), α-Terpinene (0.1%), 2-methyl-1-propenyl (0.1%), Benzene, 1-methyl-4 (0.1%), Ledene (0.1%), γ-Muurolene (0.1%), Farnesyl acetone (0.1%).	[35]
Iran	Aerial parts Comparison between 2 EOs—from wild and field conditions.	June	EOs—field conditions: (*E*)-Caryophyllene (17.813%), β-Phellandrene (14.236%), α-Humulene (10.162%), α-Pinene (5.735%), Germacrene D (5.179%). EOs—wild conditions: (*E*)-Caryophyllene (14.706%), α-Gurjunene (12.825%), Germacrene D (8.684%), α-Humulene (7.664%), β-Phellandrene (6.614%), β-Pinene (6.541%), Bicyclogermacrene (6.384%).	EOs—field conditions: β-Pinene (4.78%), Sabinene (4.538%), 1,8-Cineole (4.354%), Caryophyllene oxide (4.019%); Bicyclogermacrene (3.929%), α-Gurjunene (3.485%), Myrcene (3.025%), Spathulenol (2.844%), Linalyl acetate (1.701%), β-Bourbonene (1.562%), (*E*)-β-Ocimene (1.268%), α-Phellandrene (1.168%), Caryophylla-4(14),8(15)-dien- 5-β-ol (1.042%), Alloaromadendrene (0.965%), Linalool (0.95%), (*Z*)-β-Ocimene (0.876%), (*E*)-γ-Bisabolene (0.704%), α-Thujene (0.536%), δ-Elemene (0.518%), (*E*,*E*)-α-Farnesene (0.5%), 9-epi-(*E*)-Caryophyllene (0.406%), Camphene (0.351%), Borneol (0.351%), α-Terpineol (0.292%), γ-Terpinene (0.276%), Terpinene-4-ol (0.268%), *p*-Cymene (0.257%), *cis*-Sabinene hydrate (0.255%), β-Copaene (0.252%), β-Elemene (0.219%), Neryl acetate (0.167%), Terpinolene (0.145%), n-Nonanol (0.121%), δ-3-Carene (0.117%), α-Terpinene (0.108%). EOs—wild conditions: Eudesm-7(11)-en-4-ol (2.786%), α-Pinene (2.324%), n-Hexadecanoic acid (2.025%), Alloaromadendrene (1.708%), Caryophyllene oxide (1.557%), Myrcene (1.419%), δ-3-Carene (1.351%), Germacrene D-4-ol (1.35%), (*Z*)-β-Ocimene (1.3%), α-Cubebene (1.293%), δ-Cadinene (1.181%), (*E*)-β-Ocimene (1.033%),γ-Gurjunene (1.005%), Spathulenol (0.97%), (*E*)-γ-Bisabolene (0.944%), α-Cadinol (0.905%), δ-Elemene (0.878%), Viridiflorol (0.816%), α-Phellandrene (0.724%), Valeranone (0.694%), γ-Terpinene (0.681%), α-Copaene (0.621%), Sabinene (0.612%), Borneol (0.53%), Phytol (0.511%), γ-Cadinene (0.482%), β-Bourbonene (0.476%), Sclareol (0.415%), (*E*)-Nerolidol (0.404%), Linalyl acetate (0.383%), Camphene (0.353%), n-Pentacosane (0.334%), β-Copaene (0.324%), n-Nonanal (0.322%), α-Thujene (0.319%), β-Cubebene (0.311%), Terpinene-4-ol (0.216%), Terpinolene (0.215%), *p*-Cymene (0.199%), Alloocimene (0.188%), Linalool (0.183%), n-Tricosane (0.169%), α-Terpinene (0.145%), n-Decanal (0.139%), n-Nonanol (0.138%), *p*-Mentha-2,4(8)-diene (0.109%), *cis*-Thujopsene (0.1%).	[36]
Iran	Flowering aerial parts	July	*trans*-Caryophyllene (24.40%), β-Phellandrene (9.08%), α-Humulene (8.61%), Bicyclogermacrene (6.32%), Spathulenol (5.89%), β-Pinene (5.00%).	α-Amorphene (4.89%), Sabinene (4.44%), Limonene (3.80%), α-Gurjunene (3.32%), α-Pinene (3.03%), Myrcene (1.92%), δ-3-Carene (1.78%), (*E*)-β-Ocimene (1.68%), *(Z)*-β-Ocimene (1.65%), *trans*-Prenyl limonene (1.35%), Caryophyllene oxide (0.89%), Phytol (0.84%), β-Bisabolene (0.58%), *cis*-Calamenene (0.53%), Valeranone (0.38%), Hexahydrofarnsylacetone (0.37%), Cedranone (0.37%), α-Thujene (0.34%), α-Phellandrene (0.32%), *cis*-Cadina-1,4-diene (0.33%), Juniperol (0.30%), Terpinen-4-ol (0.28%), *trans*-γ-Bisabolene (0.27%), *ρ*-mentha-1(7),8-diene (0.24%), α-Copaene (0.25%), β-Cubebene (0.23%), α-Cubebene (0.23%), Globulol (0.22%), *ρ*-Cymene (0.22%), γ-Terpinene (0.21%), *cis*-Thujopsene (0.21%), γ-Gurjunene (0.21%), Germacrene D (0.21%), β-Sesquiphellandrene (0.21%).	[37]
Iran	Aerial parts	June	1,8-Cineole (38.26%), Camphor (22.98%), Bicycloheptan (5.52%).	Borneol (2.29%), α-Pinene (1.77%), Cyclohexane (1.67%), Camphene (0.54%).	[38]
Italy	Aerial parts Comparison between 3 EOs—aerial parts collected at different times.	September 2015-S1	S1: Germacrene D (39.5%), Bicyclogermacrene (14.8%), β-Caryophyllene (11.9%), Spathulenol (6.6%), α-Humulene (5.9%).	S1: Limonene (3.9%), β-Pinene (2.7%), (E)-β-Farnesene (2.4%), β-Bourbonene (2.1%), δ-Cadinene (1.2%).	[5]
		July 2016-S2	S2: Germacrene D (40.1%), Bicyclogermacrene (11.5%), β-Caryophyllene (7.3%).	S2: β-Phellandrene (4.9%), β-Pinene (3.7%), Spathulenol (3.1%), β-Bourbonene (3.1%), α-Humulene (2.7%); (E)-β-Ocimene (2.6%), (Z)-β-Ocimene (2.3%), (E)-β-Farnesene (1.7%), α-Pinene (1.3%), Myrcene (1.1%), Caryophyllene oxide (1.0%).	
		September 2016-S3	S3: Germacrene D (40.7%), Bicyclogermacrene (14.4%), β-Caryophyllene (7.3%).	S3: β-Phellandrene (4.5%), Spathulenol (4.2%), (E)-β-Ocimene (3.7%), (Z)-β-Ocimene (2.9%), α-Humulene (2.7%), β-Pinene (2.6%), β-Bourbonene (1.8%), (E)-β-Farnesene (1.5%), Myrcene (1.1%), Caryophyllene oxide (1.0%), α-Pinene (1.0%).	
Romania	Aerial parts	No data	β-Caryophyllene (16.03%), Caryophyllene oxide (15.24%), α-Caryophyllene (14.54%), Spathulenol (8.64%).	Terpenil acetat (3.63%), Germacrene D (2.29%), γ-Elemene (2.47%), *trans* β-Ocimen (1.95%), Patchoulol (1.77%), Limonene (1.75%), Isoaromadendrene oxide (1.67%), Isolongifolol (1.54%), *τ*-Neurolol (1.30%), Ledenoxide (0.93%), Phytol (0.79%), Hexahydroxy-farnesyl-acetone (0.72%), (*Z*)-β-Farnesene (0.46%), γ-Cadinene (0.36%), β-Pinene (0.33%), γ-Muurolene (0.30%), α-Pinene (0.29%), α-Bourbonene (0.20%), Sabinene (0.17%), *cis* β-Ocimen (0.17%), Borneol (0.17%), Borneol acetat (0.17%), Myrcene (0.16%), Ocimene (0.16%).	[39]
Iran	Aerial parts Comparison between 3 EOs—aerial parts collected from different locations.	June	Loc. 1: (E,E-α)-Farnesene (22.7–29.1%), (E)-Caryophyllene (6.7–15.5%), Bicyclogermacrene (8.1–8.2%), Germacrene B (5.9–6.2%).	Loc. 1: α-Humulene (2.9–5.4%), Caryophyllene oxide (3.1–4.4%), 1,8-Cineole (0.5–2.8%), Germacrene D (0.4–0.5%), Spathulenol (0.5%).	[40]
			Loc. 2: (E)-Caryophyllene (26.5–38.9%), (E,E-α)-Farnesene (10.5%), α-Humulene (10.3–15.9%) Germacrene D (6.3–11.5%).	Loc. 2: Bicyclogermacrene (1.5–3.9%), Germacrene B (0.9–3.3%), 1,8-Cineole (0.6–3.3%), Spathulenol (0.6–0.9%), Caryophyllene oxide (0.4–1.3%).	
			Loc. 3: (E)-Caryophyllene (9.8–24.2%), Spathulenol (4.5–15.3%), α-Humulene (4.8–11.5%), Germacrene D (9.93%), Bicyclogermacrene (7.0–9.1%).	Loc. 3: (E,E-α)-Farnesene (2.5–3.9%), Caryophyllene oxide (1.9–3.4%), Germacrene B (1.3–1.4%), 1,8-Cineole (0.9–2.5%).	
Iran	Aerial parts	No data	*trans*-Caryophyllene (18.82%), Germacrene D (9.49%), Spathulenol (7.53%), Sabinene (6.52%), Bicyclo [3.1.1] heptane, 6, 6-dime (6.0%), α-Caryophyllene (5.81%).	Bicyclogermacrene (2.66%), Aromadendrene (2.20%), δ-Cadinene (1.97%), Hexadecanoic acid (1.86%), α-Cadinol (1.78%), β-Myrcene (1.24%), Iso spathulenol (1.11%), 3-Cyclohexen-1-carboxaldehyde (0.93%), Docosane (0.84%), 1H-Benzocyclohepten-7-ol, 2, 3, 4 (0.82%), γ-Selinene (0.80%), β-Bourbonene (0.80%), Borneol (0.70%), γ-Gurjunene (0.69%), Ledol (0.67%), *cis*-α-Bisabolene (0.60%), 2-Pentadecanone, 6, 10, 14-trimethyl (0.57%), 1-Phellandrene (0.53%), Tau-Muurolol (0.48%), Vulgarol (0.43%), β-Elemene (0.40%), Tetradecanoic acid (0.37%), α-Copaene (0.35%), Cadina-1-4-diene (0.31%), Nonanal (0.31%), Phytol (0.29%).	[41]
Iran	Aerial parts Comparison between 3 EOs—aerial parts collected from 3 different origins.	No data	(*E*)-Caryophyllene (16.99–40.98%), Spathulenol (0.00–17.54%), α-Humulene (5.42–14.35%), Bicyclogermacrene (13.36–21.07%).	δ-Cadinene (1.1–3.14%), Linalol acetate (0.5–2.23%), 1,8-Cineol (0.33–2.48%), Limonene (0.31–2.91%), Linalol (0.26–1.28%).	[42]
Turkey	Aerial parts	August	β-Pinene (21.4%), 1,8-Cineole (16.1%), α-Copaene (5.4%), Alloaromadendrene (5.1%).	α-Gurjunene (4.6%), α-Pinene (3.3%), Hexadecanoic acid (2.7%), α-Cadinol (2.6%), Valeranone (2.5%), δ-Cadinene (2.5%), β-Caryophyllene (2.3%), β-Bourbonene (1.7%), Bicyclogermacrene (1.6%), Copaborneol (1.5%), Limonene (1.4%), Sabinene (1.2%), Myrcene (1.2%), Germacrene D (1.2%), Germacrene D-4-ol (1.2%), T-Cadinol (1.2%), γ-Muurolene (1.1%).	[43]
Iran	Stems, leaves, flowers	July	Stems: 1,8-Cineol(35.6%), β-Pinene (6.86%), n-Decane (5.22%), β-Cubabene (5.01%).	Stems: Bicyclogermacrene (4.64%), Germacrene D (4.34%), α-Cadinol (2.776%), δ-Cadinene (2.741%), Guaiol (1.827%), β-Gurjunene (1.72%), Spathulenol (1.585%), (E)-β-Ocimene (1.388%), γ-Cadinene (1.09%).	[44]
			Leaves: 1,8-Cineole (20.14%), α-Pinene (16.3%), δ-Elemene (10.38%), β-Pinene (9.13%), β-Gurjunen (5.36%).	Leaves: Germacrene D (3.703%), Bicyclogermacrene (3.087%), Spathulenol (3.05%), Ocimeneallo (2.973%), α-Cadinol (2.404%), Mintsulfide (2.105%), n-Decane (1.606%), δ-Cadinen (1.574%).	
			Flowers: β-Gurjunene (14.6%), Germacrene D (9.58%), δ-Elemene (9.0%),1,8 –Cineole (7.4%), (E)-β-Ocimene (5.65%), δ-Cadinene (5.25%).	Flowers: Ocimeneallo (3.494%), Spathulenol (3.04%), Myrcene (2.884%), α-Pinene (2.305%), γ-Cadinene (2.21%), 4-Terpineol (2.11%).	
Ukraine	Leaves	No data	Tritriacontane (15.6%), Nonacosane (11.5%).	γ-Sitosterol (2.9%), Docosane (2.4%), Heptacosane (2.3%), Hexahydrofarnesyl acetone (2.1%), Pentacosane (2.0%), Heneicosane (1.9%), Caryophyllene oxide (1.6%), Tetradecane (1.4%), Dotriacontane (1.1%), *cis*-Neophytadiene (0.9%), Dihydroactinidiolide (0.6%).	[45]
Iran	Aerial parts	June	Germacrene D (24.8%), β-Caryophyllene (24.1%), α-Cadinene (12.5%), Spathulenol (9.1%), Limonene (7.1%), γ-Terpinene (7.0%).	Bicyclogermacrene (3.9%), n-Decane (1.7%), β-Bourbonene (1.5%), α-Gurjunene (1.4%), α-Pinene (0.7%), β-Pinene (0.5%).	[3]
Turkey	Aerial parts	July	β-Pinene (23.0%), α-Pinene (21.6%), β-Phellandrene (13.0%), Limonene (11.0%), 1,8-Cineole (10.9%).	β-Myrcene (4.9%), *trans*-Caryophyllene (2.0%), α-Phellandrene (1.4%), Thujene (1.0%), *p*-Cymene (0.9%), 4-Terpineol (0.6%), γ-Terpinene (0.5%), Tetradecane (0.4%), Naphthalene (0.1%), Docosane (0.1%).	[2]
Serbia	Aerial parts Comparison between 3 EOs—aerial parts collected from 3 different origins.	August	S1: β-Phellandrene (43.9%), (E)-β-Ocimene (12.2%), (Z)-β-Ocimene (10.3%), γ-Muurolene (7.9%), Myrcene (6.0%), Sabinene (5.5%).	S1: β-Pinene (3.0%), α-Phellandrene (2.8%), α-Thujene (2.5%), α-Pinene (1.9%), (E)-Caryophyllene (0.9%), δ-3-Carene (0.7%), o-Cymene (0.5%), α-Muurolene (0.3%), α-Humulene (0.2%).	[30]
			S2: β-Phellandrene (70.4%), Myrcene (6.6%), α-Pinene (5.2%).	S2: β-Pinene (3.6%), (Z)-β-Ocimene (2.6%), Sabinene (2.4%), δ-3-Carene (2.2%), α-Thujene (1.6%), α-Phellandrene (1.4%), (E)-β-Ocimene (1.4%), (E)-Caryophyllene (1.2%), o-Cymene (0.9%).	
			S3: β-Phellandrene (55.5%), α-Pinene (21.1%), Myrcene (6.6%).	S3: β-Pinene (3.6%), α-Phellandrene (2.0%), Sabinene (1.7%), (Z)-β-Ocimene (1.7%), α-Thujene (1.6%), (E)-β-Ocimene (1.0%), o-Cymene (0.6%), (E)-Caryophyllene (0.3%).	
Iran	Aerial parts	May	β-Caryophyllene (31.5%), Germacrene D (16.2%), Limonene (15.5%), α-Pinene (10.4%), α-Humulene (9.4%).	No data	[46]
Turkey	Aerial parts, flowers	No data	Caryophyllene oxide (21.8–25.4%), Phytol (11.4%), Caryophylla-2(12),6-dien-5β-ol (=Caryophyllenol II) (10.7–13.6%), Hexahydrofarnesyl acetone (9.7–10.0%), Spathulenol (9.0–19.7%).	β-Caryophyllene (3.3%), Caryophylla-2(12),6-dien-5α-ol (=Caryophyllenol I) (3.2–3.6%), Caryophylla-2(12),6(13)-dien-5α-ol (=Caryophylladienol II) (2.5–2.6%), Perilla alcohol (1.9–2.5%), δ-Cadinene (1.3–1.6%), α-Cadinol (1.5%), Humulene epoxide-II (1.1–1.6%), *trans*-α-Bergamotol (0.9%), 1-Octen-3-ol (0.7%), Muurola-4,10(14)-dien-1-ol (0.7%), Farnesyl acetone (0.6–1.1%), 8,13-Epoxy-15,16-dinorlab-12-ene (Sclareol oxide) (0.6%), Isophytol (0.4%), Caryophylla-2(12),6(13)-dien-5β-ol (=Caryophylladienol I) (0.4–1.2%), (*E*)-β-Ocimene (0.4%), (*E*)-β-Damascenone (0.4%), 2-Pentadecanone (0.4%), Dimethyl tetradecane (0.3%), α-Terpineol (0.3%), Octacosane (0.3%), T-Muurolol (0.3%), α-Calacorene (0.3–0.4%), Tricosane (0.2–0.4%), α-Humulene (0.2–0.3%), *p*-Cymene (0.2%), Clovenol (0.2%), Bicyclogermacrene (0.2%), Calamenene (0.2%), Aromadendrene (0.2%), Humulene epoxide-I (0.2%), 3,4-Dimethyl-5-pentylidene-2(5 H)-furanone (0.2–0.3%), (*E*)-Geranyl acetone (0.2–0.6%), (*E*)-Nerolidol (0.2–0.7%), Tetracosane (0.2–0.8%), Hexacosane (0.2–1.0%), Alloaromadendrene (0.1%), γ-Cadinene (0.1%), (*Z*)-β-Farnesene (0.1%), Cuparene (0.1%), 1-Dodecanol (0.1%), α-Copaene (0.1–0.5%), (*E*)-β-Ionone (0.1–0.7%), α-Muurolene (0.1–1.0%), Terpinen-4-ol (0.1–1.3%).	[47]

**Table 3:** Biological activity of *S. verticillata* extracts—in vitro studies.

Study Objectives	Study Design	Main Results	References
*S. verticillata*, aerial parts, methanol extracts	Study on the antimicrobial activity evaluated on 8 bacterial strains and 8 fungal strains, using Müller–Hinton Broth. Study on the antioxidant effects using green phosphate/Mo (V) complex, diphenyl-1-picrylhydrazyl (DPPH) radical scavenging, ABTS radical cation scavenging activity, nitric oxide (NO) radical scavenging activity, a measurement of inhibitory activity toward lipid peroxidation, and a measurement of ferrous ion chelating ability.	Mild antimicrobial activity against all 8 bacterial strains (*B. cereus*—* MIC 1.25 mg/mL, *B. mycoides*—MIC 10.00 mg/mL, *M. lysodeikticus*—MIC 10.00 mg/mL, *A. chroococcum*—MIC 10.00 mg/mL, etc.) and less activity against fungi (except *C. albicans*—MIC 10.00 mg/mL, *P. canescens*—MIC 5.00 mg/mL). A significant antioxidant activity was observed (DPPH radical scavenging activity—* IC50: 33.04 ± 5.83 μg/mL; ABTS radical cation scavenging activity: IC50: 67.01 ± 13.62 μg/mL; NO radical scavenging activity—IC50: 73.12 ± 19.04 μg/mL; inhibitory activity toward lipid peroxidation—IC50: 58.07 ± 9.72 μg/mL; metal chelating activity—IC50: >4000 μg/mL).	[1]
*S. verticillata*,crude extract	Study on the antioxidant activity, using DPPH radical scavenging.	It was established that the antioxidant activity of *S. verticillata* crude extract is based on the flavone chrysoeriol.	[81]
*S. verticillata* subsp. amasiaca, essential oils and extracts	Study on the antioxidant activity, using DPPH radical scavenging; anticancer activity on cancer prostate (PC-3) and human glioblastoma U-87 MG cell lines and anticholinesterase activity, using Ellman’s colorimetric procedure.	The most significant antioxidant activity showed the *S. verticillata* EOs (flowers, aerial parts)—IC50: 24.52 and 18.89 µg/mL; and BuOH extracts (aerial parts)—IC50: 27.80 µg/mL. Anticholinesterase activity was also established.	[47]
*S. verticillata*, *S. przewalskii*, *S. miltiorrhiza*,methanol extracts	Investigation of the antioxidant activity by the Trolox equivalent antioxidant capacity (TEAC) assay, DPPH radical scavenging activity, and phosphomolybdenum assay (P–Mo) of three *Salvia* species from Poland.	The *S. przewalskii* extract showed a more significant antioxidant effect compared to the other species. The antioxidant activity of *S. verticillata* root and leaf extracts, using TEAC assay, was determined as 6.07 ± 0.2 mg/g; 13.30 ± 0.4 mg/g; DPPH radical scavenging activity—* EC50: 14.52 ± 2.02 mg/g; 19.84 ± 0.64 mg/g.	[82]
*S. verticillata*, *Filiendula ulmaria*, aqueous extracts	Study on the antimicrobial activity, using the microdilution method on 11 bacterial and 8 fungal strains; antioxidant activity, using DPPH and ABTS radical scavenging; and cytotoxic activity on normal human lung fibroblast MRC-5, human chronic myelogenous leukemia K562, human placental choriocarcinoma JEG-3, human breast cancer MDA-MB-231, and human colon cancer HCT-116 cell lines of *S. verticillata*, *Filipendula ulmaria* from Serbia.	Both plants (*S. verticillata*, *Filiendula ulmaria*) could be used for the synthesis of nanoparticles (NPs) with antibacterial activity mostly on *S. aureus*—MIC: 78.1 μg/mL; <39.1 µg/mL, *S. enteritidis*—MIC: <39.1 μg/mL; <39.1 µg/mL, *B. cereus*—MIC: <39.1 μg/mL; <39.1 µg/mL, *B. subtilis*—MIC: <39.1 μg/mL; 78.1 µg/mL, *E. faecalis*—MIC: <39.1 μg/mL; <39.1 µg/mL, *K. pneumoniae*—MIC: <39.1 μg/mL; <39.1 µg/mL, etc., and antifungal activity mostly on *Penicillium* moulds—MIC: < 78.1 μg/mL; 78.1 μg/mL, *T. lougibrachiatum*—MIC 312.5 μg/mL; <78.1 μg/mL, *C. albicans*—MIC 312.5 μg/mL; 312.5 μg/mL, and antioxidant activity. Cytotoxic activity was observed on HCT-116 cells after 24 h IC50: 44.62 μg/mL; 72 h IC50: 31.50 μg/mL for *S. verticillata*, and after 72 h IC50: 66.51 μg/mL for *Filiendula ulmaria*.	[83]
*S. verticillata* subsp. *verticillata*, ethanol extracts	Investigation of antifungal activity of *S. verticillata* from Turkey.	Antifungal activity is observed against *Cryptococcus laurentii*—MIC: 1.56–6.25 mg/mL, *C. neoformans*—MIC: 1.56–6.25 mg/mL, and *Geotrichum candidum*—MIC: 1.56–6.25 mg/mL.	[84]
*S. verticillata*, chloroform extracts, petroleum ether extracts	Study on *S. verticillata* antioxidant activity, using the DPPH test, ABTS test, Cupric Reducing Antioxidant Capacity (CUPRAC) test, Ferric Reducing Antioxidant Power (FRAP) assay, and Total Reducing Power (TRP) assay. Study on *S. verticillata* antimicrobial activity against 9 bacterial and 1 fungal strains; and cytotoxic activity on human breast cancer (MDA-MB-231) and human colorectal carcinoma (HCT 116) cell lines.	Antioxidant activity, antimicrobial activity of *S. verticillata*, chloroform and petroleum ether extracts against *E. coli*—MIC: 12.50/50.00 mg/mL; 6.25/25.00 mg/mL, *S. enteritidis*—MIC: 25.00/50.00 mg/mL; 6.25/25.00 mg/mL, *E. aerogenes*—MIC: 25.00/50.00 mg/mL; 6.25/25.00 mg/mL, *S. aureus*—MIC: 25.00/25.00 mg/mL; 6.25/25.00 mg/mL, *E. faecalis*—MIC: 12.50/25.00 mg/mL; 6.25/25.00 mg/mL, *B. cereus*—MIC: 6.25/25.00 mg/mL 25.00/25.00 mg/mL, etc., were established. Cytotoxic activity of chloroform extracts on MDA-MB-231 and HCT 116 cell lines (IC50: 77.16 μg/mL; 105.08 μg/mL) and petroleum ether extracts (IC50: 30.90 μg/mL; 44.28 μg/mL) was observed.	[85]
*S. aethiopis*, *S. candidissima*, *S. limbata*, *S. microstegia*, *S. nemorosa*, *S. pachystachys*, *S. verticillata*, *S. virgata*, methanol extracts	Study on the antioxidant activity of 8 *Salvia* species from Eastern Anatolia, Turkey, using DPPH assay.	The highest antioxidant activity was shown by *S. verticillata*—IC50: 18.3 μg/mL.	[86]
*S. blepharochlaena*, *S. euphratica var. leiocalycina*, *S. verticillata* subsp. *amasica*, methanol, aqueous, and dichloromethane extracts	In vitro investigation of antioxidant activity, using ABTS and DPPH methods, FRAP, CUPRAC, metal chelating, and phosphomolybdenum assays; cytotoxic activity on human breast adenocarcinoma (MCF-7) and human alveolar lung epithelial carcinoma (A549) cell lines, using MTT assay; and inhibiting enzyme activity of three *Salvia* species.	The aqueous extract of *S. verticillata* showed the highest antioxidant activity (DPPH method: 382.74 mg TE/g extract; ABTS method: 795.33 mg TE/g extract; CUPRAC: 829.08 mg TE/g extract; FRAP: 560.38 mg TE/g extract; metal chelating assay: 11.34 mg EDTAE/g extract), while the dichloromethane extract showed the highest enzyme inhibitory activity (AChE inhibition: 1.80 ± 0.11 mg GALAE/g extract; BChE inhibition: 1.75 ± 0.05 mg GALAE/g extract; amylase inhibition: 0.90 ± 0.09 mmol ACAE/g extract; glucosidase inhibition: 10.40 ± 0.26 mmol ACAE/g extract). The aqueous extract of *S. verticillata* showed the highest tyrosinase inhibition: 32.95 ± 2.21 mg KAE/g extract. Cytotoxic activity against MCF-7 and A549 cells was established by *S. euphratica var. leiocalycina* dichloromethane extract ((IC50: 44 μg/mL; 176 μg/mL).	[21]
*Salvia verticillata* L., *Salvia tomentosa*, *Phlomis lychnitis* L., ethyl acetate, methanol extracts	Study on the antioxidant activity of *S. verticillata* and *S. tomentosa* from Antalya, Turkey; *Phlomis lychnitis* from Konya, Turkey. The DPPH assay was used.	*S. verticillata* methanolic extract has the highest antioxidant activity due to the highest concentration of phenols and flavonoids—* SC50: 0.010 ± 0.000 mg/mL.	[87]
*Salvia verticillata* L., leaf and root, ethanol extracts	Study on the *S. verticillata* antioxidant activity using the FRAP and CUPRAC methods, ABTS, DPPH scavenging activity, and Fe2^+^ chelating activity.	Antioxidant activity of *S. verticillata* leaf (SvL) and root (SvR) extracts by DPPH—IC50: 40.03 ± 0.02 μg/mL; 97.94 ± 0.20 μg/mL, ABTS scavenging activity—IC50: 23.51 ± 0.01 μg/mL; 79.20 ± 0.11 μg/mL, and Fe2+ chelating activity—IC50: 139.78 ± 0.01 μg/mL; 580.04 ± 0.02 μg/mL was established.	[88]
*Salvia verticillata* L. ssp. amasiaca, Salvia albimaculata Hedge and Hub, *Salvia candidissima* Vahl. ssp. occidentalis, *Salvia aucheri Bentham* var. canescens Boiss and Heldr, *Salvia cryptantha* Montbret and Bentham, Salvia sclarea L., Salvia ceratophylla L., Salvia syriaca L., Salvia cyanescens Boiss and Bal., Salvia multicaulis Vahl., Salvia frigida Boiss, Salvia forskahlei L., Salvia migrostegia Boiss and Bal., Salvia halophila Hedge, ethyl acetate, chloroform, methanol, and petroleum ether extracts	Study on the antioxidant activity, evaluated by *Xanthine oxidase* (XO) inhibition assay and DPPH scavenging assay, and anticholinesterase activity, using a modified spectrophotometric method, of 14 *Salvia* species.	The extracts from *S. albimaculata* (petroleum ether) and *S. cyanescens* (chloroform) showed acetylcholinesterase inhibitory activity (17.2 ± 1.11%; 41.3 ± 2.02%) at 0.2 mg/mL. The extracts from *S. migrostegia* (ethyl acetate), *S. frigida*, *S. ceratophylla* and *S. candidissima* ssp. *occidentalis* (chloroform), and *S. cyanescens* (petroleum ether) showed butylcholinesterase inhibitory activity (38.2 ± 1.78%; 67.8 ± 5.23%; 57.4 ± 2.58%; 74.8 ± 2.09%; 81.3 ± 1.83%) at 0.2 mg/mL.	[89]
*S. verticillata* subsp. *amasiaca* (*SVA*), *Phlomis pungens* var. *hirta* (*PPH*), leaves and flowers, methanol extracts	Study on *SVA* and *PPH* antibacterial activity.	The *SVA* methanol extract showed inhibitory activity against *E. coli*, *St. aureus*, *Ps. aeruginosa*, *B. subtilis*, *B. cereus*, and *Salmonella enteritidis* (MIC: 50.0 mg/mL; 50.0 mg/mL; 50.0 mg/mL; 25–50.0 mg/mL; 25–50.0 mg/mL; 50.0 mg/mL). The *PPH* methanol extract showed inhibitory activity against *B. subtilis* and *Ps. aeruginosa* (MIC: 50.0 mg/mL; 50.0 mg/mL).	[79]
*S. verticillata* L. var. verticillata, S. frigida Boiss., S. russellii Benth., S. virgata Jacq., S. candidissima subsp. candidissima Vahl., seeds	Study on the antimicrobial activity of 4 bacterial, 2 fungal, and 2 dermatophyte strains, using the disc diffusion method; antioxidant activity, using DPPH and ABTS radical scavenging activity, of 5 *Salvia* species from Turkey.	The seed extracts showed antimicrobial and antioxidant activity. *S. verticillata* showed the highest activity against *S. aureus* (13.33 ± 0.3 mm), *B. megaterium* (17.33 ± 0.3 mm), and *C. albicans* (16.66 ± 0.33 mm).	[90]
*Salvia* species, ethanol, aqueous extracts	Study on the antioxidant activity of 10 *Salvia* species from Germany. The QUENCHER method was used.	The results indicate that the ethanolic extracts of *S. verticillata* and *S. forsskaolii* had antioxidant activity comparable to *S. officinalis*.	[91]
*S. officinalis* L., *S. verticillata* L., S. aethiopis L., S. glutinosa L., S. austriaca Jacq., S. nemorosa L., S. pratensis L., S. nutans L., S. ringens Sibth & Sm., methanol extracts	Study on the antioxidant activity of 9 *Salvia* species from Romania.	*S. officinalis* provided the strongest antioxidant protection, followed by *S. verticillata.*	[22]
*S. verticillata*, *S. officinalis*, *S. tesquicola*, *S. sclarea*, *S. austriaca*, *S. aethiopis*, *S. kopetdaghensis*, *S. pratensis*, *S. nutans*, *S. nemorosa*, ethanol extracts	Study on the antioxidant activity, using ABTS, DPPH, and FRAP assays; antimicrobial activity, against 4 bacterial (*S. aureus*, *S. pneumoniae*, *E. coli*, *P. aeruginosa*) and 1 fungal (*C. albicans*) strains; and cytotoxic activity on human breast carcinoma MCF-7 and MDA-MB-231 cell lines of 10 *Salvia* sp. from Moldova.	The results showed significant antioxidant and antimicrobial activity in *Salvia* species, led by *S. officinalis*. No cytotoxic activity was observed against breast cancer cell lines. MIC values of *S. verticillata* are as follows: *S. aureus*—1.25 mg/mL, *S. pneumoniae*—2.5 mg/mL, *E. coli*—2.5 mg/mL, *P. aeruginosa*—2.5 mg/mL, and *C. albicans*—2.5 mg/mL.	[92]
*S. verticillata* L., methanol extract	In vitro examination of *S. verticillata* antibacterial activity and antioxidant activity, using DPPH scavenging assay.	Antioxidant activity (IC50: 0.61) and antimicrobial activity against *E.coli* (94.86% of dead cells) and *Listeria innocua* (97.77% of dead cells) of methanol extracts of *S. verticillata* have been proven.	[80]
*S. officinalis* L., *S. verticillata* L., S. virgata Jacq., S. reuterana Boiss., S. hypoleuca Benth., ethanol extracts	Investigation of antioxidant activity of 5 *Salvia* species. The DPPH scavenging assay was used.	The results showed that these species have an antioxidant activity close to the standard *S. officinalis* (IC50: 23.53–125.1 μg/mL), the highest being for *S. verticillata* (23.53 (20.56–26.93) μg/mL).	[93]
*S. officinalis*, *S. verticillata*, *S. fruticosa*, *S. nemorosa*, *S. glutinosa*, *S. sclarea*, *S. pratensis*, ethanol extracts	Study on the antioxidant activity, using DPPH, Reducing power, and lipid peroxidation inhibition assays; NO radical scavenging and iron chelating activity; hypoglycaemic and neuroprotective activity of 7 *Salvia* species.	The results showed that these *Salvia* species inhibit alpha-glucosidase and acetylcholinesterase. *S. verticillata* showed acetylcholinesterase inhibitory activity with IC50: 1607.87 ± 15.05 μg/mL. The ethanol extracts also possess antioxidant activity—DPPH (IC50: 2.49–7.71 μg/mL); lipid peroxidation inhibition assays (IC50: 53.18; 116.83–327.23 μg/mL); NO radical scavenging (IC50: 26.96–101.73 μg/mL); iron chelating activity (*S. sclarea* IC50: 163.02 μg/mL; *S. officinalis*, *S. verticillata*, *S. fruticosa* IC50: 1185.54–1582.53 μg/mL); and *S. verticillata* acetylcholinesterase inhibition—IC50: 1607.87 ± 15.05 μg/mL.	[94]
*Salvia* species, extracts	Examine of antioxidant activity of 60 *Salvia* species from Anatolia.	All species have shown high antioxidant activity.	[95]
*S. trichoclada*, *S. suffruticosa*, *S. multicaulis*, *S. euphratica*, *S. candidissima* subsp. *candidissima*, *S. russellii*, *S. microstegia*, *S. verticillata* L. subsp. verticillata, *S. virgata*, *S. frigida*, *S. ceratophylla*, *S. aethiopis*, seeds, n-hexane extracts	Investigation of antimicrobial activity of 12 *Salvia* species.	The results showed that the extracts have variable antibacterial activity against *Staphylococcus aureus*, *Escherichia coli*, *Klebsiella pneumoniae*, *Candida glabrata*, *Candida albicans*, *Bacillus megaterium*, *Epidermophyton* sp., and *Trichophyton* sp.	[96]
*S. verticillata*, *Zataria multiflora*, *Froriepia subpinnata*, ethanol extracts	Study on the antimicrobial activity of *S. verticillata*, *Zataria multiflora*, *Froriepia subpinnata* against *Pseudomonas aeruginosa*, and *Pectobacterium carotovorum*.	The ethanolic extract of *Zataria multiflora* showed the highest antimicrobial activity (*Zataria multiflora*—MIC: 3.12–6.25 mg/mL; *S. verticillata*—MIC: 12.3–25 mg/mL; *F. subpinnata*—MIC: 12.5–25 mg/mL). Of the possible combined extracts, the most effective is the combination of *Salvia verticillata* and *Froriepia subpinnata*.	[97]
*S. verticillata*, alcoholic/aqueous extract	Study of *S. verticillata* antioxidant and neuroprotective activity.	The aqueous extract of *S. verticillata* positively affected the viability of rat pheochromocytoma cell lines.	[98]
*S. officinalis*, *S. verticillata*, *S. sclarea*, *S. przewalski*, *S. ringens*, *S. jurisicii*, *S.pratensis*, *S. nemorosa*, *S. hians*, *S. nemorosa var. haemathodes*, *Salvia x superb*, methanol extracts	Study on the insecticidal activity of *Salvia* species on *Spodoptera littoralis*.	The methanol extracts of *S. hians* and *S. przewalskii* showed the highest insecticidal activity on *Spodoptera littoralis* (total mortality 80.9%, 81.5%).	[99]
*S. verticillata*, *S. trichoclada*, *S. fruticosa*	In vitro investigation of the antioxidant activity, using DPPH scavenging assay, and the inhibitory ability of *S. verticillata*, *S. trichoclada*, and *S. fruticosa* extracts on acetylcholinesterase, using the Ellman method.	All extracts demonstrated antioxidant activity. *Salvia trichoclada* (methanolic extract) showed the highest inhibitory ability on acetylcholinesterase: ±81.10% at 2 mg/mL concentration.	[100]

* MIC—minimum inhibitory concentration; * IC50—half-maximal inhibitory concentration; * EC50—half-maximal effective concentration; * SC50—half-maximum stimulating concentration.

**Table 4:** Biological activity of *S. verticillata* EOs—in vitro studies.

Study Objectives	Study Design	Main Results	References
*S. verticillata* EO from Iran.	Study on the cytotoxic activity.	A cytotoxic activity was observed against cell lines of colon adenocarcinoma (Caco-2—IC50: 125.12 ± 27.59 μg/mL; HT-29—IC50: 90.90 ± 14.88 μg/mL) and breast ductal carcinoma (T47-D—IC50: 80.20 ± 8.91 μg/mL), with higher effect on colorectal adenocarcinoma.	[37]
*Salvia dicroantha*, *S. verticillata* subsp. *amasiaca* and *Salvia wiedemannii* from Turkey.	Study on the antimicrobial, antifungal, and anticholinesterase activity.	Antimicrobial activity against Gram-negative and Gram-positive microorganisms has been established, and antifungal activity against *Candida glabrata*, *Candida albicans*, and *Saccharomyces* *Cerevisiae* (MICs: 12.5–50.0 µL/mL). Only *S. wiedemannii* essential oil demonstrated inhibitory activity against butylcholinesterase (50.97 ± 3.12%) and acetylcholinesterase (55.95 ± 2.01%).	[106]
*S. verticillata*, EO from Georgia.	In vitro study on the antioxidant activity, using the Oxygen Radical Absorbance Capacity (ORAC) assay, and anti-inflammatory activity, tested on produced NO by mouse macrophage cells (RAW264.7).	Anti-inflammatory activity (inhibition of NO production by RAW264.7 for methanol fraction: 100%; chloroform fraction-83%) and antioxidant activity (ORAC assay for aqueous fraction: 6.7 ± 0.3 μmol/TE/mg) were established.	[108]
*Salvia aramiensis*, *Salvia aucheri* subsp. *aucheri*, *Salvia fruticosa*, *Salvia tomentosa and S. verticillata* L. subsp. amasiaca EOs from Turkey.	Study on the activity against *Mycobacterium tuberculosis*, using the MGIT fluorometric manual method.	EOs of *S. aucheri* subsp. *aucheri* (196.0 μg/mL), *S. tomentosa* (196.0 μg/mL), and *S. verticillata* subsp. *amasiaca* (196.0 μg/mL) showed antimycobacterial activity.	[43]
*S. verticillata* L., Stachys lavandulifolia Vahl., Tanacetum polycephalum Schultz-Bip. EOs from Iran.	Study on the antibacterial activity.	*S. verticillata* (MIC: 1.23 mg/mL) and *Stachys lavandulifolia* (MIC: 2.15 mg/mL) EOs showed greater effectiveness against *Escherichia coli*, while *Tanacetum polycephalum* (MIC: 1.00 mg/mL) EO showed it against *Staphylococcus aureus.*	[41]
*S. verticillata*, *S. sclarea*, *S. limbata*, *S. multicaulis*, *S. choloroleuca* EOs from Iran.	In vitro investigation of the antibacterial activity, using the disc diffusion method (DDM) and the minimum inhibitory concentration (MIC) method.	EOs from *Salvia* species showed greater efficacy against Gram-positive bacteria than Gram-negative bacteria and no activity against *K. pneumoniae*. *S. verticillata* showed activity against *S. aureus* (MIC: 15) and *E. coli* (MIC: 15).	[107]
*S. verticillata* L., S. nemorosa L., S. aethiopis L. EOs from Romania.	Study on the antibacterial activity, using Muller–Hinton agar (MHA) and microplates methods.	The results showed activity against *Staphylococcus aureus* (5–20% EO concentration) compared to *Escherichia coli*. The microplates method showed that *S. verticillata* EO MIC was rated at 0.5%.	[39]
*S. verticillata*, *S. multicaulis*, and *S. sclarea* aerial parts EOs from Iran.	Study on the antibacterial activity, using the DDM and 11 bacterial strains.	*S. verticillata*, *S. multicaulis*, and *S. sclarea* EOs showed antibacterial activity against *S. aureus*, *S. epidermidis*, *B. pumulis*, *B. subtilis* (MIC: 3.75–7.5 mg/mL), *E. coli*, *K. pneumoniae*, (MIC: 15.0 mg/mL; >15 mg/mL), etc., and no activity against *Ps. aeruginosa*.	[46]
*S. verticillata* ssp. *amasiaca*, *S. macroclamys*, *S. virgata*, *S. firigida*, *S. multicaulis*, *S. kronenburgii*, *S. microstegia* EOs from Turkey.	Study on the antibacterial and antifungal activity, using the agar diffusion test.	Low antibacterial activity against some bacteria and fungi (*S. aureus*—8 mm, *E. coli*—8 mm, *K. pneumonia*—6 mm, *C. albicans*—10 mm), and no activity against *Pseudomonas aeruginosa*.	[2]

**Table 5:** Biological activity of *S. verticillata*—in vivo studies.

Study Objectives	Study Design	Main Results	References
*S. verticillata*, hydro-alcoholic extract	Study of *S. verticillata* antioxidant activity in mice.	An improvement is observed in the states of depression and seizures.	[109]
*S. verticillata*, ethanol extracts	Study of *S. verticillata* hypoglycaemic activity on rats for 14 days.	*S. verticillata* ethanol extracts increased the levels of insulin and decreased the levels of glucose, having also renal protective and hepatoprotective effects in a concentration-dependent manner.	[110]
*S. fruticosa*, *S. verticillata*, and *S. trichoclada*, methanol, aqueous, chloroform, acetone, and n-butanol extracts	Investigation of the anti-inflammatory activity on rats of *S. fruticosa*, *S. verticillata*, and *S. trichoclada* from Turkey.	A positive effect on inflammation was observed from all three species of *Salvia* due to the phenolic acids, flavonoids, and terpenoids in the composition. *S. fruticosa* extract (n-butanol) has a significantly greater anti-inflammatory effect. *S. verticillata* extracts at concentrations of 50–100 mg/kg showed anti-inflammatory activity 1.4–5.6% (1 h), 3.8–12.00% (2 h), 1.78–26.79% (3 h), 2.7–18.3% (4 h).	[4]
*S. verticillata*, alcoholic extract	Study of the antioxidant activity of *S. verticillata* on 24 rats with cerebral hypoperfusion for 14 days.	A reduction in oxidative stress-related damage was observed.	[111]
*S. verticillata* L., *S. patens* L., aqueous/alcoholic extracts	Examination of the anti-inflammatory activity and acute toxicity of *S. verticillata* and *S. patens* in rats for 14 days.	The extracts showed a moderate anti-inflammatory effect and no toxicity.	[112]

## Data Availability

Data are contained within the article.
